# Supplementary material for: WordCommentsAnalyzer: A windows software tool for qualitative research
Source: F1000Res. 2018 Sep 4;7:536. Originally published 2018 May 3. [Version 2] doi: 10.12688/f1000research.14819.2 (PMC6137411; doi:10.12688/f1000research.14819.2)
Supplement: Supplementary file 3 [file f1000research-7-17614-s0002.tgz › b2a79496-4ada-47c4-9682-a99ce867836d.zip!/Eight Iranian Health Journals/Journal of Research in Rehabilitation Sciences _25.docx]

© Journal of Research in Rehabilitation Sciences . This work is licensed under CC BY-NC 4.0 (https://creativecommons.org/licenses/by-nc/4.0/).

Volume & Issue: ویژه نامه ورزشی: 1392

Title: اثر سطوح مختلف فعالیت ورزشی بر تعادل ایستا و پویای پای برتر و غیر¬برتر زنان

http://jrrs.mui.ac.ir/index.php/jrrs/article/view/1261

مقدمه: هدف از تحقیق حاضر، اثر سطوح مختلف فعالیت ورزشی بر تعادل ایستا و پویای پای­برتر و غیر­برتر زنان ورزشکار و غیرورزشکار بوده است. مواد و روش‌ها: آزمودنی‌های تحقیق حاضر را 24 زن ورزشکار فوق‌فعال با میانگین سن 35/1±4/22 سال، قد 10/6±5/164 سانتیمتر و جرم 35/5±46/56 کیلوگرم، 24 زن ورزشکار فعال با میانگین سن 71/0±08/22 سال، قد 37/5±75/159 سانتیمتر و جرم 48/5±76/59 کیلوگرم و 24 زن غیرورزشکار با میانگین سن 21/1±20/21 سال، قد 38/4±96/160 سانتیمتر و جرم 78/5±14/59 کیلوگرم تشکیل دادند که به صورت داوطلبانه در این تحقیق شرکت کردند. افراد فوق‌فعال و فعال هفته‌ای حداقل 6 و 3 جلسه به ترتیب و هر جلسه یک تا یک و نیم ساعت به فعالیت ورزشی می‌پرداختند. اما افراد غیرفعال نیز شامل کسانی بود که فاقد هرگونه فعالیت ورزشی منظمی بودند. آزمودنی‌ها هیچ گونه آسیب‌دیدگی در اندام تحتانی، آسیب سر و سیستم وستیبولار نداشتند. از آزمون لک‌لک برای ارزیابی تعادل ایستا (پایای 87/0) و از آزمون ستاره برای ارزیابی تعادل پویا (98/0-94/0 ICC= - 96/0-85/0ICC=) استفاده شد. از آنالیز واریانس یک طرفه و آزمون تعقیبی توکی برای بررسی تفاوت معناداری بین هر کدام از گروه‌ها و از t وابسته نیز برای بررسی تفاوت درون‌گروهی استفاده شد. یافته‌ها: نتایج تحقیق حاضر تفاوت معناداری را بین زنان فوق‌فعال، فعال و غیرفعال در تعادل ایستا و پویا، به صورت جداگانه، نشان داد (05/0P≤ ) در حالی که تفاوت معناداری بین تعادل پای برتر و غیربرتر افراد در تمام گروه‌های حاضر مشاهده نشد (05/0P≥ ). نتیجه گیری: براساس نتایج تحقیق حاضر، بنابراین، به افراد غیرفعال توصیه می‌شود که جهت بالا رفتن تعادل که جزء مهمی از زندگی محسوب می‌شود، در فعالیت‌های ورزشی مشارکت فعال داشته باشند.

Title: تأثير سرعت‌هاي مختلف تصويرسازي ذهني حرکت بر عملکرد

http://jrrs.mui.ac.ir/index.php/jrrs/article/view/1281

مقدمه: هدف تحقیق حاضر بررسي تأثير سرعت‌هاي آهسته و واقعي تصويرسازي ذهني حرکت بر عملکرد بود. مواد و روش‌ها: بدين منظور 30 شرکت کننده در سه گروه تصويرسازي با سرعت آهسته، تصويرسازي با سرعت واقعي و کنترل بدون تصويرسازي تخصيص داده شدند. تکليف شامل دريبل توپ فوتبال بود. بعد از انجام پيش آزمون، سه جلسه تمرين تصويرسازي حرکت (هر جلسه 30 کوشش) انجام شد. بعد از جلسه سوم تصويرسازي آزمون يادداري انجام شد. یافته‌ها: يافته‌ها نشان داد که هر دو نوع تصويرسازي با سرعت آهسته و زمان واقعي منجر به کاهش زمان اجرا شد، ولي تفاوتي در خطاي اجرا مشاهده نشد.  بحث: با توجه به نتايج تحقيق اثر همگون سازي مورد تأييد قرار نگرفت. به نظر مي‌رسد در افراد مبتدي کاهش سرعت تصويرسازي منجر به تمرکز توجه بيشتري روي مؤلفه‌هاي شناختي حرکت و در نتيجه بهبود اجرا شده بود.

Title: تفاوت‌های الگوی حرکتی ناحیه کمری-لگنی در دو گروه از افراد کمردردی دارا و فاقد فعالیت‌هاي چرخشی طی آزمون فعال چرخش خارجی مفصل ران

http://jrrs.mui.ac.ir/index.php/jrrs/article/view/1211

مقدمه: هدف از مطالعه حاضر مقایسه ی الگوی حرکتی ناحیه کمری-لگنی بین دو گروه افراد کمر دردی دارا و فاقد فعالیت‌های نیازمند چرخش، طی آزمون فعال چرخش خارجی ران بود. مواد و روش‌ها: در مطالعه حاضر 39 بیمار مبتلا به کمردرد مزمن غیر اختصاصی شامل، 15 آزمودنی (میانگین سنی 5/31) دارای فعالیت‌های نیازمند چرخش مانند تنیس، اسکواش و ... و 24 آزمودنی فاقد فعالیت‌های نیازمند چرخش شرکت داده شدند. داده‌های کینماتیکی ناحیه کمری-لگنی-ران آزمودنی‌ها طی آزمون فعال چرخش خارجی مفصل ران توسط سیستم آنالیزر حرکتی با قابلیت سه بعدی سازی جمع‌آوری شدند. متغیرها شامل دامنه چرخش خارجی ران، دامنه چرخش خارجی اندام تحتانی، چرخش لگن، دامنه چرخش لگن در نیمه اولیه چرخش ران و همزمانی حرکت لگن با مفصل ران توسط برنامه MATHLAB محاسبه شد. سپس از آزمون t-test مستقل به منظور مقایسه متغیرهای مشابه بین دو گروه استفاده گردید. یافته‌ها: چرخش کمری-لگنی در گروه بیماران دارای فعالیت‌های نیازمند چرخش به طور قابل توجه بیشتر از گروه دیگر بود (03/0=p). اما بین دو گروه تفاوت قابل ملاحظه‌ای در دیگر متغیرها وجود نداشت (05/0=p). نتیجه گیری: الگوی حرکتی ناحیه کمری-لگنی گروه‌های بیماران مبتلا به کمر درد با توجه به تکالیف اختصاصی تکراری هر گروه، متفاوت با سایر گروه‌های بیمار می‌باشد.

Title: مقايسه ي شدت درد،سطح ناتواني عملكردي،پاسخ هاي ترس-اجتناب از حركت و ميزان فعاليت هاي تفريحي در دو گروه از بيماران مبتلا به كمردرد مزمن غير اختصاصي.

http://jrrs.mui.ac.ir/index.php/jrrs/article/view/1179

چكيده: زمينه و هدف:عوامل مختلفي در تعيين ميزان ناتواني عملكردي بيماران مبتلا به كمردرد نقش دارند.در اين ميان به نظرميرسيد سطح ترس و اجتناب ازحركت از طريق كاهش سطح فعاليت هاي فيزيكي افراد، نقش مهمي در افزايش ميزان ناتواني بيماران مبتلا به كمردردايفا مي كند.هدف از اين مطالعه مقايسه ي ميزان ناتواني وسطح ترس-اجتناب از حركت افراد مبتلا به كمردرد مزمن غيراختصاصي داراي فعاليت هاي ورزشي اختصاصي وافراد مبتلابه كمردرد مزمن غير اختصاصي فاقد فعاليت هاي ورزشي اختصاصي مي باشد. مواد و روش ها:در اين مطالعه 39 مرد مبتلا به كمردرد مزمن غير اختصاصي(15 نفر داراي فعاليت هاي ورزشي اختصاصي و24نفر فاقد فعاليت هاي ورزشي اختصاصي)شركت داشتند.شدت دردكمر،ميزان ناتواني هاي عملكردي ،سطح پاسخ هاي ترس-اجتناب از حركت وسطح فعاليت هاي تفريحي آنهاثبت و دردو گروه با يكديگر مورد مقايسه قرار گرفتند. يافته ها:در گروه داراي فعاليت هاي ورزشي اختصاصي ميزان ناتواني هاي عملكردي،سطح ترس-اجتناب از حركت به طور معنادار كمتر از گروه فاقد فعاليت هاي اختصاصي ورزشي بود(p</05)و اين درحالي است كه سطح فعاليت هاي تفريحي در گروه داراي فعاليت هاي ورزشي اختصاصي به طور معناداربيشتر از گروه فاقد فعاليت هاي اختصاصي ورزشي مي باشد. (p=/00) بين دو گروه تفاوتي در ميزان شدت درد كمر ديده نشد.(p=/00) نتيجه گيري:شركت در فعاليت ورزشي و تفريحي مي تواند از طريق كاهش پاسخ هاي ترس و اجتناب از حركت، ميزان ناتواني هاي عملكردي بيماران مبتلا به كمر درد مزمن غير اختصاصي را كاهش دهد.همچنين تفاوت بين گروههاي مبتلا به كمردرد مي تواند نشان دهنده ي ضرورت تقسيم بندي بیماران مبتلا به کمردرد در گروههاي همگن باشد.

Title: بررسی تأثیر ورزشهای ثباتی گردن دربهبودجلوآمدگی سر در دانشجویان دختر27-20 ساله دانشگاه علوم پزشکی شیراز

http://jrrs.mui.ac.ir/index.php/jrrs/article/view/1358

مقدمه : یکی ازمشکلات شایع در جوامع کنونی، اختلال در راستای ستون فقرات گردنی بوده و جلوآمدگی سر شایع‌ترین اشکال در راستای ناحیه گردن می‌باشد. هدف از این تحقیق بررسی تأثیر ورزش‌های ثباتی گردن در بهبود جلوآمدگی سر می‌باشد. مواد و روش‌ها: 26 دختر دانشجوی دانشگاه علوم پزشکی شیراز که مشکل جلوآمدگی سر داشتند با محدوده سنی 27-20 سال دراین تحقیق کارآزمایی بالینی شرکت کردند. افراد مورد مطالعه به صورت تصادفی به دو گروه مورد وشاهد تقسیم شدند. لندمارک‌های موردنیاز برای اندازه گیری جلوآمدگی سر شامل تریگوس و زائده خاری مهره هفتم  گردنی توسط مارکرهای سفید مشخص گردید. پس از اندازه‌گیری میزان جلوآمدگی سر، گروه مداخله به مدت 8 هفته ورزش‌های ثباتی گردن را انجام داده و سپس اندازه زاویه جلو آمدگی سر در هر دوگروه مقایسه گردید.  یافته ها: نتیجه حاصل از مقایسه میانگین اختلاف زاویه جلوآمدگی سر، قبل و بعد از مداخله در گروه مورد نشان داد این زاویه به طور معنی‌داری نسبت به گروه کنترل کاهش داشته است ( 05/0< P). نتیجه گیری: ورزش‌های ثباتی گردن باعث کاهش زاویه جلوآمدگی سر می‌گردد. بنابراین می‌تواند در بهبود جلو آمدگی سر مؤثر باشد.

Title: مقایسه نسبت قدرت عضلانی و دامنه حرکتی چرخش داخلی به خارجی مفصل شانه هندبالیست های نخبه آسیب دیده و سالم

http://jrrs.mui.ac.ir/index.php/jrrs/article/view/1389

مقدمه: مفصل شانه یکی از آسیب پذیرترین مفاصل بدن در ورزشکاران هندبال است. تکرار زیاد حرکات پرتابی می تواند باعث تغییر تعادل قدرت عضلانی و دامنه حرکتی شانه ورزشکاران می شود. با توجه اینکه تعادل قدرت و دامنه حرکتی در پیشگیری از آسیب­های شانه  اهمیت دارد. بنابراین، هدف از انجام این تحقیق مقایسه نسبت قدرت عضلانی و دامنه حرکتی چرخش داخلی به خارجی دست غالب و غیرغالب هندبالیست های سالم و آسیب دیده است. مواد و روش‌ها: آزمودنی‌های این تحقیق شامل 68 بازیکن هندبال بودند که با فرم ثبت آسیب به دو گروه سالم (سن:82/3±85/23؛ شاخص توده بدن:05/2±19/23؛ سابقه بازی: 22/3±82/10) و آسیب دیده (سن:43/4±27/24 ؛ شاخص توده بدن:08/2±32/23؛ سابقه ورزشی:90/3±27/11) تقسیم شدند. برای ازریابی قدرت عضلات از دینامومتر دستی  و برای ارزیابی دامنه حرکتی مفصل شانه از انعطاف سنج جاذبه ای لیتون استفاده شد. یافته‌ها: بر اساس یافته های تحقیق بین نسبت قدرت چرخش داخلی و خارجی و دامنه حرکتی اندام غالب هندبالیست های سالم وآسیب دیده  به ترتیب (004/0=p؛ 001/0≥p) اختلاف معنا دار وجود دارد. اما در اندام غیرغالب اختلاف معنا داری(32/0=p؛ 9/0=p) مشاهده نشد .همچنین نتایج اختلاف معنی داری را در نسبت قدرت و دامنه حرکتی در اندام غالب و غیر غالب بازیکنان آسیب دیده به ترتیب(007/0=p؛ 01/0≥p) نشان داد. علاوه براین نتایج اختلاف معنا داری را در نسبت قدرت و دامنه حرکتی اندام غالب و غیرغالب بازیکنان سالم نشان داد به ترتیب(001/0≥p؛ 04/0=p). نتیجه‌گیری: انجام حرکات تکراری پرتاب از بالای سر در طولانی مدت باعث تغییر در تعادل قدرت عضلات و دامنه حرکتی مفصل می شود، بنابراین برنامه ریزی برای کنترل و محدود کردن این ریسک فاکتور باید در دستور کار مربیان و عوامل پزشکی تیم قرار گیرد.

Title: تأثیر فعالیت هوازي در آب و خشکی برروی بتا اندورفين پلاسما و آستانه ادراك درد ورزشكاران

http://jrrs.mui.ac.ir/index.php/jrrs/article/view/1334

چکیده   مقدمه: فعالیت ورزشی با تغییر در میزان مصرف انرژی باعث کنش دستگاه های مختلف بدن از جمله سیستم عصبی- هورمونی و غدد درون ریز می شود. هدف از مطالعه حاضر بررسی تاثیر فعالیت هوازی در آب و خشکی بروی میزان بتااندورفین و آستانه درد ورزشکاران می باشد. مواد و روش ها: ۱۷دختر والیبالیست (با حداقل سه سال سابقه فعالیت مداوم و میانگین سنی 1۲/4±52/۲۷ سال، وزن 29/5±44/۶۱ کیلوگرم و قد 86/4±59/۱70 سانتی متر)، بصورت داوطلبانه در این تحقیق شرکت کردند. آستانه درد و نمونه­گیری خونی از سیاهرگ بازویی قبل، بلافاصله و ۳۰ دقیقه پس از فعالیت هوازی طی دو جلسه در محیط آب و خشکی، اندازه گیری شد. تجزیه و تحلیل آماری داده ها با استفاده از روش آماری اندازه گیری های مکرر(Repeated measurement)، و برای تعیین جفت مقایسه ها از آزمون تعقیبی LSD استفاده شد. همچنین جهت مقایسه سطح بتا اندورفین و آستانه درد در دو محیط آب و خشکی برای مراحل بلافاصله پس از آزمون و پیگیری، پس از محاسبه نمرات افزوده، از آزمون تی همبسته (paired t test)  استفاده شد. یافته ها: طبق نتایج بدست آمده بین میزان بتا اندورفین و آستانه ادراک درد، قبل و بعد از فعالیت ورزشی تفاوت معنی دار وجود داشت، بطوری که فعالیت هوازی منجر به افزایش معنادار بتا اندورفین و نیز آستانه ادراک درد در هر دو محیط شد (۰۰۰۱/۰>P)،(۰۰۱/۰>P). میزان بتا اندورفین خون دختران ورزشکار پس از فعالیت هوازی در آب، در مقایسه با خشکی به‌ طور معناداری کمتر بود. این تفاوت به طورمعناداری 30 دقیقه پس از فعالیت نیز مشاهده شد (۰۱/۰>P). علاوه براین، بین آستانه ادراک درد در شرایط پس از فعالیت هوازی درآب، با شرایط مشابه در خشکی تفاوت معناداری وجود نداشت(۳٤/۰P=). در مرحله 30 دقیقه پس از فعالیت نیز در مقایسه تمرین هوازی در موقعیت‌های مختلف نیز تفاوت معناداری در آستانه ادراک درد مشاهده نشد (56/0P=). نتیجه گیری: فعالیت هوازی در هر دو محیط آب و خشکی منجر به افزایش بتااندورفین و آستانه درد می شود. بنظر می رسد، تغییرات در آستانه درد ناشی از فعالیت هوازی، مستقل از محیط فعالیت است، اما در مورد تغییرات بتا اندورفین ناشی از تمرین، اثرات محیط معنادار است.
